# PB.05: MRI-guided vacuum-assisted breast biopsy at 3T: initial experience

**DOI:** 10.1186/bcr3507

**Published:** 2013-11-08

**Authors:** M Sreenivas

**Affiliations:** 1 University Hospitals Coventry and Warwickshire NHS Trust, Coventry, UK

## Introduction

As the specificity is relatively low, histological confirmation of incidental breast lesions at 3T is needed if the treatment plan has to change. MRI-guided biopsy is needed as a proportion of these lesions will not been seen on conventional imaging.

## Methods

Forty-nine out of 240 patients undergoing DCE-MRI studies performed between 1 July 2011 and 20 July 2013 warranted second-look US (20% of patients). Fifteen of the 49 patients underwent MRI-guided biopsy predominantly as second-look US was negative. Diagnostic imaging and biopsy were performed on GE 3T using a dedicated breast coil, grid method, CADstream software and Vacora vacuum-assisted biopsy device with a 10G needle obtaining between 10 and 22 cores.

## Results

Fourteen of the 15 patients had technical success (in one patient the biopsy had to be performed twice due to unsatisfactory sampling, and repeat biopsy yielded B5b).

Seven nonmass M3 lesions yielded a 43% malignancy rate (B4 = 1 and B5a = 2). Two of five M4 lesions were masses yielding B5a with overall malignancy yield in this category of 40%. One of the two M5 masses was B5b with a cancer yield in this category of 50%. Overall malignancy yield was 43%.

## Conclusion

As far as we know we are the only unit in the UK to perform MRI-guided breast biopsy at 3T and our results are in accordance with published literature (cancer yield between 24 and 40%). This preliminary work has shown that MRI biopsy at 3T is feasible using a hand-held multiple-insertion vacuum-assisted device.

